# The influences of perceived green advertising value and motivation types on green purchasing intention

**DOI:** 10.3389/fpsyg.2026.1766561

**Published:** 2026-04-17

**Authors:** Thu Ha Bui, Fumikazu Morimura, Kimitaka Nishitani

**Affiliations:** 1 Graduate School of Business Administration, Kobe University, Kobe, Japan; 2 Research Institute for Economics and Business Administration, Kobe University, Kobe, Japan

**Keywords:** advertising value, green advertising, green purchase intention, motivation, self-determination theory

## Abstract

This study investigates how consumers' perceived green advertising values influence different motivation types and—in turn—their green purchase behavioral intention. By using the Stimulus-Organism-Response model and self-determination theory, we examine perceived green advertising value's effects on intrinsic motivation (personal satisfaction) and four extrinsic motivation regulations (external regulation/social approval, introjected regulation/guilt or obligation, identified regulation/personal values, and integrated regulation/identity alignment) and these motivations' influences on green purchase intention. We analyze a statistically representative sample of 439 Japanese consumers through a partial least square structural equation modeling (PLS-SEM). Our findings highlight the importance of various motivational factors in mediating the relationships between perceived green advertising value and green purchase intention, in which intrinsic motivation and integrated regulation emerged as strong mediators. Our results also underscore the importance of perceived green advertising value in shaping consumers' motivation. Regarding different motivation types' effects on green purchase intention, we find a lack of impact of introjected regulation. Integrated regulation emerges as the strongest predictor of green purchase intention. We draw out implications and outline future research directions from the study findings.

## 1 Introduction

Growing awareness of climate change, pollution, and resource depletion is driving consumers to consider the environmental impacts of their purchases, leading to increased demand for sustainable, environmentally friendly products across industries. According to the United Nations Sustainable Development Goals (SDGs), SDG 12 emphasizes responsible consumption and production patterns, aiming to minimize toxic waste by 2030. Meanwhile, according to the 2024 Sustainable Development Goals Report, SDG12 is only 40% on track, while 60% has stalled or regressed (United Nations, 2024). In Japan, efforts to promote ethical consumption that considers people, society, and the environment face challenges. In the 2024 Ethical Consumption Awareness Survey, conducted nationwide, only 27.4% of respondents knew the term, and 36.1% reported practicing it, with the highest rates among those aged 70 and older (Consumer Affairs Agency, 2024). This situation highlights the urgent need for collaboration between stakeholders and effective green communication strategies to boost sustainable practices.

Green advertising is a common communication method used by marketers to position products or services as environmentally friendly (Eren-Erdogmus et al., 2016). It includes advertisements that explicitly or implicitly address the relationships between a product/service and its biophysical environment, promote green lifestyles, or present a company as environmentally responsible (Banerjee et al., 1995). By emphasizing both a product/service and its environmental benefits, green advertising highlights a company's commitment to sustainability and encourages consumers to choose environmentally friendly products. Scholars of green marketing have concluded that advertising influences consumer behaviors when they perceive advertising value (Wei et al., 2021; Bi et al., 2023). Ducoffe (1995) proposed the advertising value construct to measure consumers' perceptions regarding advertising's relative worth or utility. Key factors that shape this value include informativeness, entertainment, irritation and credibility (Van-Tien Dao et al., 2014; Firat, 2019; Disastra et al., 2019). While studies have linked advertising value to consumer responses such as attitudes, purchase intention, advertisement avoidance and involvement (Firat, 2019; Gaber et al., 2019; Wei et al., 2021; Hussain et al., 2023), we still lack research that explores it in the green purchase context.

Besides the advertising value, this study focuses on consumer motivations to understand why consumers do not buy environmentally friendly products. Motivation is the reason upon which one acts, and is a key antecedent to behaviors (Graafland and de Bakker, 2021). Motivation—a fundamental mechanism that connects individuals' values with their pro-environmental behaviors—has been extensively investigated across diverse domains, including green infrastructure (Silvi and Padilla, 2021), employees' green behaviors (Norton et al., 2015). In the green advertising context, Lima et al. (2024) argue that green advertising's effectiveness cannot be studied without considering consumers' intrinsic motivation to act sustainably; they call for further investigation of the relationships between green marketing, intrinsic motivation and behaviors. Duong et al. (2023) indicate that a pronounced imbalance between green extrinsic and intrinsic motivations will undermine individuals' engagement in environmentally sustainable consumption behaviors. However, previous research has primarily focused on extrinsic motivation such as rewards and incentives, while it has neglected the diverse motivational categories proposed by Self-Determination Theory (SDT): external control/social approval, internal control/guilt or obligation, identified control/personal values, and integrated control/identity consistency (Ng et al., 2024). Further, the application of self-determination theory to green advertising has remained underexplored, particularly in examining how green advertising evokes specific motivation types.

We seek to fill these gaps in the literature in the green marketing and consumer behaviors contexts by examining the influences of perceived green advertising value on different motivation types toward green purchases. By incorporating SDT into the Stimulus-Organism-Response (S-O-R) model of green advertising, we reveal that the “organism” is not a single latent state but rather a structured pathway of internalization, thereby adding process-level insights to the literature on green advertising. To contribute to the study of consumers' green purchase decision-making processes, we asked:

RQ1: What is the effect of perceived green advertising value on various motivational constructs?

RQ2: In what ways do distinct motivation types contribute to increasing green purchase intention?

Based on recognized gaps in existing research, we offer substantial contributions to the green purchasing literature. First, we enhance the understanding of the motivational drivers in green purchasing by clarifying how different motivation types mediate the relationships between perceived green advertising value and green purchasing intention. Second, we extend the research by combining perceived green advertising value and both intrinsic and extrinsic motivations in the green purchasing context, based on the S-O-R model and SDT. Third, we employ a representative sample of 439 Japanese consumers, adding contextual relevance, since most of the previous studies were done either in Western contexts or in China. Along Hofstede's cultural dimensions, Japan scores high on long-term orientation and uncertainty avoidance, which is reflected in consumer behaviors that prioritize sustainability and clear information and that reduce risks. Thus, Japan is a representative research field in which to examine the value and motivations of perceived green advertising. Studying the influences of perceived green advertising value and motivation types on green purchase intention in Japan can offer valuable cross-cultural insights.

The following sections are structured as follows: In Section 2 we summarize the relevant literature based on the S-O-R model, SDT, findings and gaps in the literature. In Section 3 we develop hypotheses based on the insights gained from the literature review. In Section 4 we describe our research methodology and the procedures we used, including the information on how we collected the data as well as the measurement items we used for analysis. In Section 5 we present the data analysis' results. In Section 6, we discuss our research findings. In Section 7 we summarize our contributions, the study limitations, and potential future research directions.

## 2 Theoretical background

### 2.1 The stimulus-organism-response model

The S-O-R model was proposed by Mehrabian and Russell (1974) is used to explain the relationships between environmental stimuli (*S*), organisms (*O*) and behavioral outcomes (*R*). According to this framework, external environmental stimuli influence consumer behavior indirectly; they first trigger the consumer's internal state, which subsequently leads to behavioral responses.

The S-O-R model is widely adopted in consumer behavior research. The green consumption literature on the S-O-R model includes many stimuli, including marketing communication sources, advertising appeal and consumer confidence (Sultan et al., 2021; Wang et al., 2021; Li and Shan, 2025). To reflect organisms, attitude has been widely used (Sultan et al., 2021). However, green consumption can be viewed as an ethical practice, making the motivations to engage in its critical factors in driving sustainable behaviors (Wang et al., 2024, 2021). Our review finds a lack of research into motivation as internal state.

In the green advertising context, Goyal et al. (2026) noted that the S-O-R model is among the most frequently used theories alongside attribution theory, elaboration likelihood model. Whereas much green advertising work relies on attribution theory to explain skepticism and greenwashing, S-O-R is highlighted as a prominent mechanism-oriented framework. Building on this, Li and Shan (2025) employ S-O-R to show how green perceived value and green trust mediate the effect of green advertising receptivity on purchase intention, while we extend the organism component by modeling how perceived green advertising value shapes distinct motivational states that, in turn, drive green purchase intention.

### 2.2 Self-determination theory and motivations

Formulated by Deci and Ryan (1985), SDT offers a perspective on human motivation and personality by incorporating both empirical methodologies and an organismic meta-theoretical lens. This approach underscores the role of innate psychological capacities in fostering personality development and self-regulation (Ryan and Deci, 2000). SDT posits that human motivation and behavior are fundamentally shaped by the satisfaction of three basic psychological needs: autonomy, competence, and relatedness.

**Figure 1:**
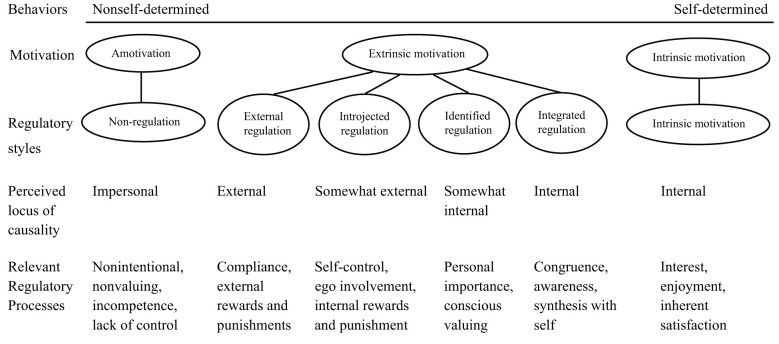
Motivation types and subtypes in self-determination theory (Ryan and Deci, 2000, p.72).

SDT categorizes motivations along self-determination levels into three types: intrinsic motivations, extrinsic motivations and amotivation (Figure 1). Amotivation refers to a state characterized by an absence of control and feelings of detachment or disconnection, often conceptualized as learned helplessness. Intrinsic motivation is natural inclinations to engage in an activity for the inherent satisfaction derived from the activity itself, guided by personal interest and choice, encouraging us to pursue activities that resonate with our inner values. In contrast, extrinsic motivation occurs when activities are performed to gain positive outcomes or avoid negative consequences, and has four distinct types: external, introjected, identified and integrated regulation.

According to SDT, all the motivational (sub)types are arranged along a continuum based on their associated self-determination level (Ryan and Deci, 2000). External regulation describes behaviors governed by incentives and penalties imposed from outside the individual, resulting in actions that are perceived as controlled rather than autonomous. Introjected regulation refers to extrinsic motivations that have been partially internalized; behaviors are regulated by the internal rewards of self-esteem for success or the avoidance of negative emotions like anxiety, shame, or guilt in the event of failure. In identified regulation, a person personally accepts the significance of an activity and acts with a sense of volition and purpose. Integrated regulation concerns conditions in which individuals fully incorporate the value of an activity, perceiving it as consistent with their core values and interests (Ryan and Deci, 2000).

In the marketing communications context, intrinsic and extrinsic motivations have been used as independent variables as well as mediating and moderating variables to predict consumer behavioral outcomes such as purchase intention (Gilal et al., 2019, 2022). Motivations have been recognized as key mediating factors, facilitating the translation of personal traits, needs or environmental influences into actual behaviors. Studies have shown this effect: message tailoring and framing—motivation—behaviors (Pelletier and Sharp, 2008); advertising appeal—intrinsic motivation—purchase intention (Wang et al., 2021). Motivation can also function as a moderator, since the strengths and directions of marketing messages' impacts on behaviors depend on the type and level of motivation present in an individual. Gilal et al. (2020) found that external motivation has a stronger effect on brand passion for men and older customers, while intrinsic motivation is more influential for women and younger customers, which suggests that the marketing effectiveness varies depending on whether consumers are more internally or externally motivated as well as on demographic factors.

In the green purchasing context, Wang et al. (2024) incorporated the extended theory of planned behavior and SDT to investigate green purchase behaviors in China, revealing that intrinsic motivation positively affects green purchase intention. Ng et al. (2024) integrated customer value theory and SDT, finding that product values (emotional value, functional value and relational value) predict four regulation types (external regulation, introjected regulation, identified regulation and integrated regulation). Although these studies have made significant contributions to the green purchasing literature, there are limitations owing to the research scope and the data. Further, the application of SDT in the marketing communication context, especially green advertising, has remained underexplored. We examine the influences of intrinsic motivation and the four regulations of extrinsic motivation on green purchase intention, which is driven by consumers' perceptions of green advertising value.

## 3 Hypothesis development

### 3.1 Advertising value and motivations

Ducoffe (1995) proposed the advertising value construct to measure consumers' perceptions of advertising's relative worth or utility. Ducoffe (1995) identified three primary antecedents that contribute to perceived advertising value, including informativeness (the extent to which an advertisement provides useful information to a consumer), entertainment (the extent to which an advertisement is enjoyable and engaging) and irritation (the negative feelings or annoyance that can arise from an advertisement). These antecedents directly influence consumers' attitudes toward advertisements. A positive perception of advertising value can lead to favorable attitudes, while high irritation can diminish this value (Zhang et al., 2024). The higher perceived advertising value of an advertisement increases the motivation to buy a product. This is because high-value advertisements capture attention, create positive attitudes, and create meaningful connections with consumers' needs or values (Disastra et al., 2019; Hussain et al., 2023).

#### External regulation

External regulation refers to a psychological state in which an individual is driven by the desire to gain externally imposed rewards or to avoid externally enforced punishments. Green advertising's effectiveness in promoting extrinsic motivation is supported by findings suggesting that consumers are more likely to respond positively when they perceive that their purchasing choices contribute to broader social goals or comply with normative standards. In other words, extrinsic motivation refers to regulation driven by contingent outcomes, such as social approval or conformity to others' expectations, and does not refer to the specific content of green advertising. Green advertising may evoke social pressures and expectations, thereby encouraging consumers to make greener choices. Elgaaied-Gambier et al. (2018) found that social descriptive norms influence intention to purchase non-overpackaged products indirectly, through advertising credibility. Thus, we hypothesize:

H1: Perceived green advertising value is positively associated with external regulation.

#### Introjected regulation

Introjected regulation reflects internalized self-esteem processes, including the avoidance of guilt and shame or the pursuit of pride. Message frames in green advertising (gain vs. loss) can trigger consumer attention, thus evoking emotional responses that align with introjected regulation. Baek and Yoon (2017) emphasized the interplays between negative emotions and message framing in environmental advertisements, while Amatulli et al. (2019) found that anticipated shame is the key emotion underlying the superior effectiveness of negatively framed messages, compared to positively framed ones, in encouraging pro-environmental behavior. Thus, we hypothesize:

H2: Perceived green advertising value is positively associated with introjected regulation.

#### Identified regulation

Identified regulation refers to a state in which an individual engages in specific behaviors they perceive as personally meaningful and purposeful, even if these actions are not inherently enjoyable. In the green advertising context, green cues that highlight a product's environmental features lead people to place greater social and ethical value on green products than on conventional ones, as they are not merely purchasing a product but fulfilling their own value (Haytko and Matulich, 2008; Santa and Drews, 2023). The following hypothesis is proposed:

H3: Perceived green advertising value is positively associated with identified regulation.

#### Integrated regulation

Integrated regulation occurs when individuals internalize a behavior, making it a fully aligned and congruent part of their identity, and incorporating its execution into their sense of self. Consumers who appreciate green advertising often see themselves as environmentally conscious individuals. This identification creates a sense of alignment between their actions and their self-image, enhancing their motivation to engage in sustainable practices. Yang et al. (2025) indicated that green self-identity enhances the positive relationship between green marketing practices and customer loyalty within the new energy vehicle market. A study on green advertising's post-purchase effects by Meijers et al. (2019) showed that those with stronger environmental identity are more inclined to maintain pro-environmental behaviors. Thus:

H4: Perceived green advertising value is positively associated with integrated regulation.

#### Intrinsic motivation

Intrinsic motivation is characterized by an inherent propensity to pursue novelty and challenges, to enhance one's skills, and to explore and learn (Ryan and Deci, 2000). Individuals driven by intrinsic motivation engage in an activity for its inherent interest and satisfaction. Lima et al. (2024) indicated that intrinsic pro-environmental motivation plays a key role in the persuasive process from exposure to a green message to pro-environmental intention and action. Wang et al. (2021) showed that, when consumers are exposed to organic appeals in advertisements, they exhibit higher intrinsic motivation, which in turn influences their intention to purchase organic products. Therefore:

H5: Perceived green advertising value is positively associated with intrinsic motivation.

### 3.2 Motivations and green purchase behaviors

#### External regulation

External regulation refers to a psychological state in which individuals are driven by a desire to gain externally imposed rewards or to avoid externally enforced punishments. Some studies indicated that social factors, such as peer influences and collectivism, along with cognitive factors such as perceived behavior control and subjective norms, have strong positive impacts on green purchase intention (Ajzen, 1991; Suki and Suki, 2019). Therefore:

H6: External regulation positively influences green purchase intention.

#### Introjected regulation

Introjected regulation involves internal pressures on self-esteem, for example, avoiding guilt and shame or pursuing pride. Haj-Salem et al. (2022) found that anticipated guilt positively influences the intention to purchase green products. According to Mukherjee and Chandra (2022), anticipated guilt shapes individual thinking and future ecological choices by fostering positive attitude and greater environmental concern. Grounded in these findings, we propose:

H7: Introjected regulation positively influences green purchase intention.

#### Identified regulation

Identified regulation occurs when individuals engage in behaviors that they personally value and find meaningful, regardless of whether such acts are inherently enjoyable. In online purchasing contexts, identified regulation, along with consumer satisfaction, can optimize purchase intention. This suggests that, when consumers identify with the value of a product or service, they are more likely to make repeat purchases (Azizah and Amin, 2023). In the green consumption context, identified regulation strongly predicts green purchase intention, highlighting its role in promoting sustainable consumer behaviors (Ng et al., 2024). We hypothesize:

H8: Identified regulation positively influences green purchase intention.

#### Integrated regulation

Integrated regulation occurs when individuals internalize a behavior, making it a fully aligned and congruent part of their identity, and incorporating its execution into their sense of self. Ng et al. (2024) found that autonomous motivation, including identified regulation and integrated regulation, significantly predicts green purchase intention. An energy consumption study by Webb et al. (2022) indicated that, when consumers internalize energy-saving behaviors as part of their identity, they are more likely to act on their intention. Personal identity, as opposed to social identity, strengthens the positive influence of aligned self-transcendent intentions on green consumption behaviors (Costa Pinto et al., 2016). Therefore:

H9: Integrated regulation positively influences green purchase intention.

#### Intrinsic motivation

Pelletier et al. (1998) posited that motivational types with higher self-determination lead to more positive psychological and behavioral outcomes. Intrinsic motivation's role in driving green consumption behaviors has been investigated in the green marketing context. Duong et al. (2023) found that a significant imbalance between green extrinsic and intrinsic motivations reduces environmentally friendly consumption. Intrinsically motivated consumers have positive feelings toward green products, which underpin enjoyment in using green products and love for green brands. These enjoyment and brand love increase green purchase intention Lin, (2022). Buil and Mata (2024) revealed the moderator role of intrinsic motivation in consumer's eco shopping basket. Furthermore, Wang et al. (2021) showed that intrinsic motivation mediates the relationship between organic advertising and purchase intention, with consumers exposed to organic milk advertisement demonstrating higher intrinsic motivation and increased intention to buy organic milk products. Thus:

H10: Intrinsic motivation positively influences green purchase intention.

**Figure 2:**
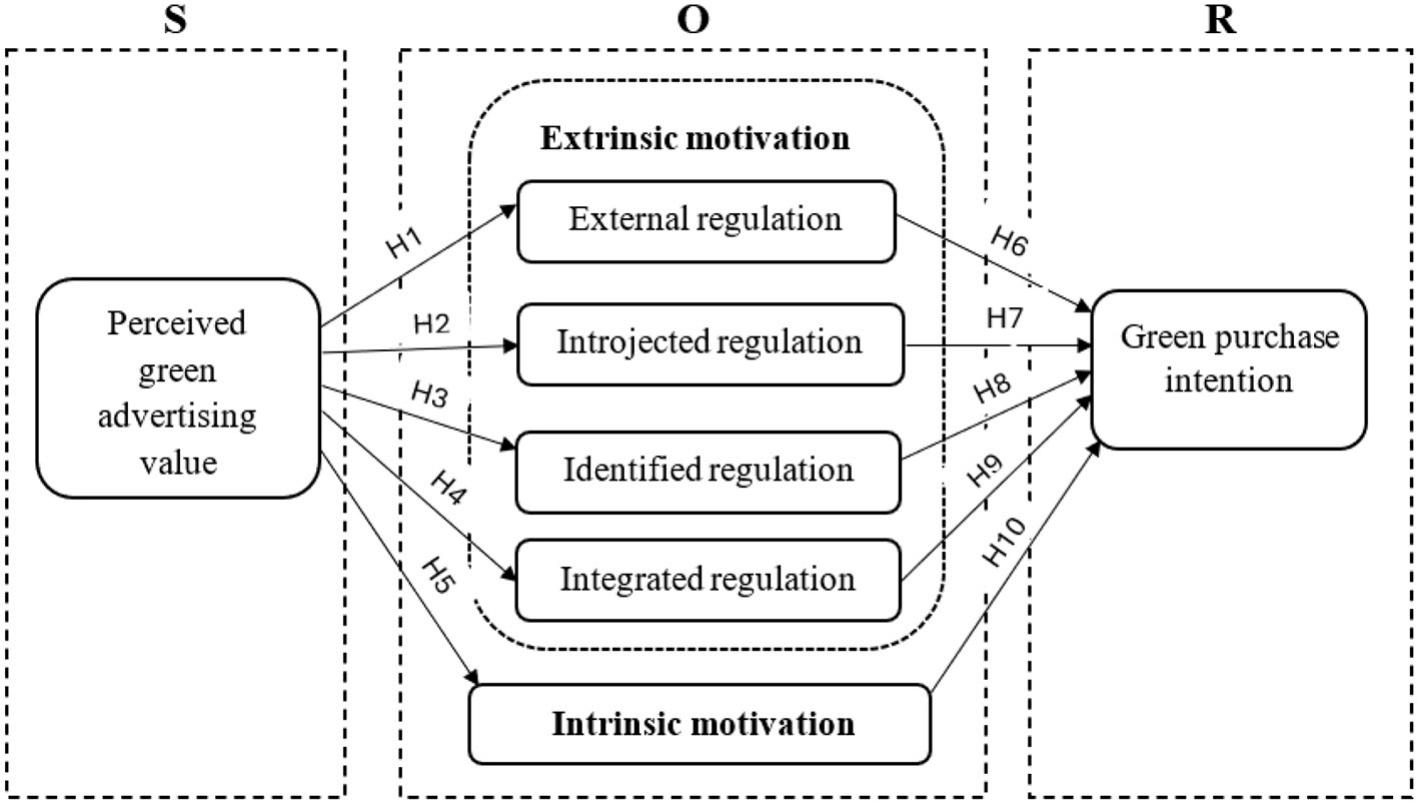
Research model.

Considering the contextual background, literature review and hypotheses formulated, we proposed a research model (Figure 2).

## 4 Methodology

### 4.1 Data collection

We collected data using the questionnaire-based survey method. The survey was conducted targeting Japanese consumers. Therefore, the survey items were translated into Japanese through parallel-translation, back-translation, and pre-test to maintain conceptual accuracy and consistency (Brislin, 1980). The survey items were translated into Japanese by three Japanese individuals fluent in both Japanese and English. We then conducted a pre-test to identify potential problems with understanding the questionnaire items. Through this translation and pre-test procedures, we created the final version of the questionnaire for data collection.

An online survey targeting Japanese participants was sent on November 27, 2024 via the Yahoo! Crowdsourcing platform, a service operated by Yahoo Japan Corporation. With around 1 million users, this platform allowed us access to a wide range of Japanese consumers. Users could see the survey's description and precautions before deciding to take part. Users who agreed to the survey objectives and precautions proceeded to the survey and received PayPay Points as compensation upon completion. The participants were adults (aged 20 or older) living in all Japan's prefectures. For the inclusion criteria, they were asked about their experiences purchasing green products and viewing green advertisements. Those fitting the specified criteria were invited to complete the online questionnaire. To minimize item context and consistency effects, the order of all items in the questionnaire was randomized. From 450 initial responses, after excluding responses that had the same rating for all questions, 439 suitable responses were received. Table 1 briefly describes a sample of the respondents.

**Table 1:** Demographics of the sample.

Variable	Value	Research sample	
		*N*	%
**Sex**	Female	199	45.3
	Male	240	54.7
**Age**	< 25	17	3.9
	25 to 34	89	20.3
	35 to 44	132	30.1
	45 to 54	102	23.2
	>55	99	22.6
**Monthly income (JPY)**	< 150k	158	36.0
	150k−300k	163	37.1
	300k−500k	74	16.9
	500k−1,000k	27	6.2
	1,000k−2,000k	3	0.7
	>2,000k	14	3.2
**Education level**	High, secondary or vocational school	74	16.9
	Professional training college	66	15.0
	Bachelor degree	235	53.5
	Master's or Ph.D.	32	7.3
	Other	32	7.3

### 4.2 Measurement

The questionnaire's constructs and items were adapted from established latent variables with validated psychometric properties. All items were measured using a five-point Likert scale ranging from 1 (strongly disagree) to 5 (strongly agree). The survey had two parts: Section 1 gathered demographic details such as gender, age, monthly income, and education level, while Section 2 included measurement items covering perceived green advertising values, motivation types and green purchase intention. The survey instruments were adapted from the literature, such as perceived green advertising value (four items) (Mustafi and Hosain, 2020; Martins et al., 2019); external regulation (four items), introjected regulation (three items), identified regulation (four items), integrated regulation (four items) (Pelletier et al.,1998; Ng et al., 2024); intrinsic motivation (four items) (Pelletier et al.,1998); green purchase intention (three items) (Hussain et al., 2023).

In this study, perceived green advertising value was measured with four items (“useful,” “valuable,” “important,” “helps to make better selections”) to capture the extent to which consumers see green advertising as a meaningful decision aid—namely, a helpful input for making purchase decisions in the environmental domain (useful; helps to make better selections), a beneficial and worthwhile communication source (valuable), and a relevant element in the marketplace (important). This construct goes beyond informational value by incorporating perceived importance and overall worth, and it implicitly reflects decision-support effectiveness (helping consumers make better selections), but it does not directly assess credibility or entertainment, which are considered potential antecedents rather than components of the construct. Compared with traditional advertising value scales that were developed for advertising in general or specific media formats and focused on general advertising attributes (e.g., informativeness, entertainment, irritation), our operationalization is domain-specific to green advertising and emphasizes whether green advertising supports better green purchase decisions rather than how entertaining or pleasant the advertisements are.

### 4.3 Data analysis

We used Partial Least Square Structural Equation Modeling (PLS-SEM) to analyze the measurement model and the structural model in the conceptual model. The SEM method can analyze all model variables simultaneously rather than individually, enabling a more precise assessment than other methods such as regression analysis. PLS-SEM is particularly effective for complex models that include multiple constructs and relationships. PLS-SEM treats the construct as a composite based on total variance, formed linearly from a set of indicator variables. As recommended by Hair et al. (2019), PLS-SEM should be selected when the goal of analysis is to test a theoretical model from a predictive standpoint; when the structural model is complex with numerous constructs, indicators or relationships. It is also suitable for exploratory research aimed at extending established theories to better understand increasing complexity. We employed PLS-SEM following Hair et al. (2019, 2021) guidelines since the model represents an exploratory extension of the S-O-R framework with SDT theory in a novel context (Japanese consumer - green advertising and aims to predict hypothetical relationships, and PLS-SEM is particularly suited to this objective as it emphasizes the interplay between prediction and theory testing with validation of results. Thus, we employed PLS-SEM for theory extension and prediction purposes.

In this study, PLS-SEM methodology has two components: the measurement model and the structural model. We assessed the constructs' validity and reliability by the indicators' outer loadings, Cronbach's alpha, composite reliability, average variance extracted (AVE) and heterotrait-monotrait ratio (HTMT) value. We then estimated the parameters of the structural model, such as path coefficients, to evaluate the hypotheses. PLS-SEM was performed using SmartPLS 3. We chose a significance level of 1% for the complexity of the involved variables.

## 5 Results

### 5.1 Common method bias

Before performing PLS-SEM, we conducted Harman's single-factor test to examine whether a single latent factor accounts for the majority of variance among the study variables. The results reveal that a single factor accounts for 47.26% of the total variance, which is below the recommended threshold of 50%.

In addition, a full collinearity test shows that all the inner VIF values are less than 3.3, so the common method bias is not a concern in our analysis. In line with recommended procedural remedies to reduce common method biases in survey research, we randomized the order of questionnaire items to reduce item context and consistency effects, thereby lowering the risk of common method bias (Podsakoff et al., 2003).

### 5.2 Measurement model

To assess the measurement model, first, we examined the indicators' outer loadings. High outer loadings on a construct indicate that the associated indicators share much in common with it. As an empirical standard, outer loadings of 0.708 or higher are desirable (Hair et al., 2021). All our item loadings exceed this threshold (Table 2).

**Table 2:** Measurement Items.

**Constructs**		Items	Factor loadings
**Perceived green advertising value (PAV)**	PAV1	I feel that green advertising is useful.	0.858
	PAV2	I feel that green advertising is valuable.	0.911
	PAV3	I feel that green advertising is important.	0.901
	PAV4	I feel that green advertising helps to make better selections.	0.867
**External regulation (EXT)**	EXT1	Other people will be upset if I don't buy green products.	0.839
	EXT2	My friends insist that I purchase green products.	0.811
	EXT3	I purchase green products for the recognition from others.	0.817
	EXT4	I purchase green products to avoid being criticized about not doing it.	0.741
**Introjected regulation (INTRO)**	INTRO1	I would regret if I didn't buy green products.	0.892
	INTRO2	I would feel guilty if I didn't purchase green products.	0.905
	INTRO3	I would feel bad if I didn't purchase green products.	0.878
**Identified regulation (IDEN)**	IDEN1	Green purchasing is a sensible thing to do.	0.833
	IDEN2	Green purchasing is a way I've chosen to contribute to the environment.	0.820
	IDEN3	Green purchasing is a reasonable thing to do.	0.841
	IDEN4	Green purchasing is a good idea to do for the environment.	0.824
**Integrated regulation (INTG)**	INTG1	Green purchasing is an integral part of my life.	0.860
	INTG2	When I purchase green products, I can take care both of myself and environment.	0.786
	INTG3	Green purchasing has become a fundamental part of who I am.	0.866
	INTG4	Green purchasing is part of the way I've chosen to live my life.	0.893
**Intrinsic motivation (IN)**	IN1	It's my pleasure to master new ways to help the environment by purchasing green products.	0.900
	IN2	It's my pleasure to improve the quality of the environment by purchasing green products.	0.916
	IN3	I feel good when doing things for the environment by purchasing green products.	0.887
	IN4	It's my pleasure to contribute to protecting the environment by purchasing green products.	0.915
**Green purchase intention (GPI)**	GPI1	I would consider purchasing green products with green advertising.	0.918
	GPI2	I intend to purchase green products with green advertising.	0.947
	GPI3	I would probably buy green products with green advertising.	0.938

Second, we checked the Cronbach's alpha, which measures internal consistency reliability. The alpha criterion provides an estimate of reliability based on the intercorrelations between the observed indicator variables. All the values in each construct were above the threshold of 0.70 (see Table 3). According to Hair et al., 2021, one weakness of Cronbach's alpha is that it assumes that all indicators are equally reliable, and it is sensitive to the number of items in a scale. Owing to the limitations of Cronbach's alpha, in PLS-SEM, composite reliability is applied to measure internal consistency reliability. Hair et al. (2021) suggested that composite reliability values between 0.7 and 0.9 can be regarded as satisfactory. All our values were within this range (see Table 3).

**Table 3:** Reliability and validity.

Measurement indicators	PAV	EXT	INTRO	IDEN	INTG	IN	GPI
Cronbach's alpha	0.907	0.821	0.873	0.849	0.874	0.926	0.927
Composite reliability	0.935	0.879	0.921	0.898	0.914	0.947	0.954
AVE	0.783	0.645	0.796	0.688	0.727	0.819	0.873
VIF	1.000	2.237	2.694	2.274	3.168	2.608	

Regarding convergent validity, the average variance extracted (AVE) exceeded the threshold of 0.5 for all constructs (see Table 3), showing that they explain more than 50% of the variance for their items (Hair et al., 2021). To accurately assess discriminant validity, we used the heterotrait-monotrait ratio (HTMT) of the correlations. The HTMT approach estimates the true correlation between two constructs if they were perfectly measured. Our results reveal that all HTMT values were below 0.90, meeting the threshold (Henseler et al., 2015) (see Table 4).

**Table 4:** Discriminant validity (HTMT ratio).

Constructs	EXT	GPI	IDEN	IN	INTG	INTRO	PAV
EXT							
GPI	0.598						
IDEN	0.415	0.751					
IN	0.400	0.770	0.819				
INTG	0.699	0.862	0.715	0.743			
INTRO	0.857	0.589	0.478	0.434	0.782		
PAV	0.452	0.768	0.749	0.720	0.722	0.454	

We also calculated variance inflation factors (VIFs) for the constructs to confirm the suspicion of multicollinearity. As all the VIF values were less than 5 (see Table 3), there are no collinearity issues among the constructs (Hair et al., 2021).

### 5.3 Structural model

#### 5.3.1 Evaluation of structural model

The evaluation of the structural model (see Table 5) comprises several criteria, namely the coefficients of determination (*R*^2^), predictive relevance (*Q*^2^), and effect sizes (*f*^2^) (Hair et al., 2021).

**Table 5:** Result of R^2^, adjusted R^2^ and Q^2^.

Constructs	*R^2^*	*R* ^2^_adj_	*Q^2^*
EXT	0.172	0.170	0.100
INTRO	0.171	0.169	0.129
IDEN	0.435	0.433	0.296
INTG	0.422	0.420	0.297
IN	0.439	0.437	0.356
GPI	0.696	0.693	0.600

*R*^2^ values of 0.75, 0.50, and 0.25 for endogenous latent variables are considered substantial, moderate, and weak, respectively (Hair et al., 2021). The results show that green purchase intention explains over 69% of the variance of motivation types and green advertising value. Similarly, identified and integrated regulation, along with intrinsic motivation, explain over 42% of the variance in green advertising value.

Predictive relevance (*Q*^2^) was assessed using the blindfolding procedure in PLS-SEM. According to established benchmarks, *Q*^2^ values of 0.02, 0.15, and 0.35 indicate small, medium, and large predictive relevance, respectively, of an exogenous construct for a given endogenous construct Hair et al., 2021). Thus, the construct cross-validated redundancy results (*Q*^2^ values in Table 4) indicate that the model's constructs exhibit predictive relevance.

The effect size (*f*^2^) is a useful component in the evaluation of the strength of a statistical claim. The f^2^ values of 0.02, 0.15 and 0.35 represent small, medium, and large effects, respectively. (Hair et al., 2021). The *f*^2^ values in Table 5 predominantly fall between the medium and large thresholds, indicating substantively meaningful relationships.

#### 5.3.2 Hypothesis test results

**Figure 3:**
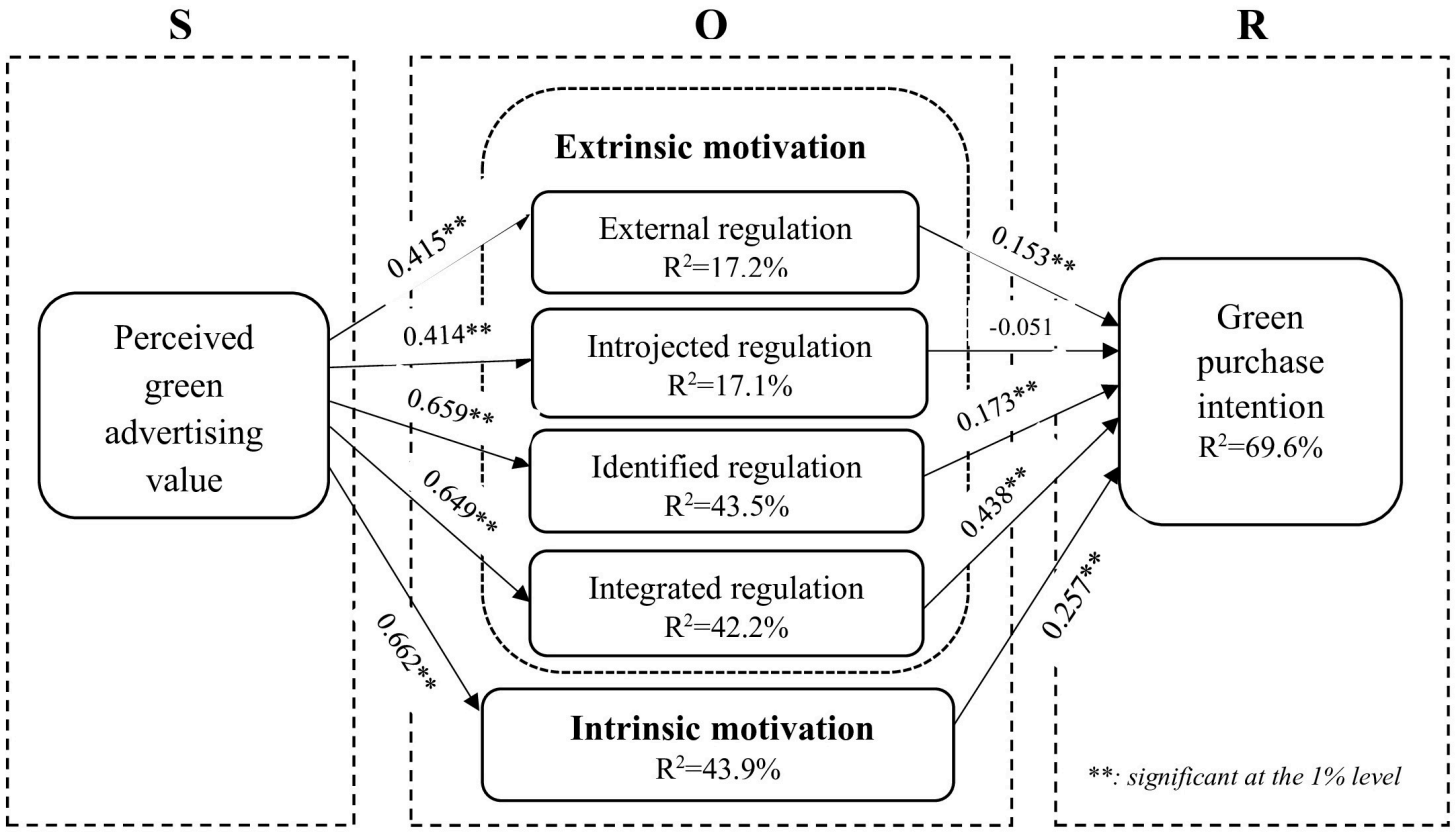
PLS-SEM results of the structural model.

We calculate the path coefficients and the *t*-values using a bootstrap analysis with 5,000 random re-samples. As shown in Table 6, perceived green advertising value has positive impacts on external regulation β = 0.415, *p*-value < 0.01), introjected regulation (β = 0.414, *p*-value < 0.01), identified regulation (β = 0.659, *p*-value < 0.01), integrated regulation (β = 0.649, *p*-value < 0.01) and intrinsic motivation (β = 0.662, *p*-value < 0.01), supporting H1 to H5. As external regulation has positive impact on green purchase intention (β = 0.153, *p*-value < 0.01), H6 is supported. As introjected regulation is not positively associated with green purchase intention (β = −0.051, *p*-value>0.01), H7 is not supported. As identified regulation is positively associated with green purchase intention (β = 0.173, *p*-value < 0.01), H8 is supported. As integrated regulation is positively associated with green purchase intention (β = 0.438, *p*-value < 0.01), H9 is supported. As intrinsic motivation is positively associated with green purchase intention (β = 0.257, *p*-value < 0.01), H10 is supported. The results are presented in Figure 3.

**Table 6:** Summary of the PLS-SEM analysis.

Path	Hypothesis	Path coefficients	Standard deviation	*f^2^*	*t*-statistics	*p*-values	Conclusion
PAV–> EXT	H1	0.415	0.044	0.208	9.441	0.000	Supported
PAV–> INTRO	H2	0.414	0.041	0.207	10.127	0.000	Supported
PAV–> IDEN	H3	0.659	0.034	0.769	19.465	0.000	Supported
PAV–> INTG	H4	0.649	0.030	0.729	21.691	0.000	Supported
PAV–> IN	H5	0.662	0.032	0.782	20.952	0.000	Supported
EXT–> GPI	H6	0.153	0.040	0.034	3.792	0.000	Supported
INTRO–> GPI	H7	−0.051	0.042	0.003	1.209	0.227	Not supported
IDEN–> GPI	H8	0.173	0.055	0.043	3.167	0.002	Supported
INTG–> GPI	H9	0.438	0.050	0.200	8.731	0.000	Supported
IN–> GPI	H10	0.257	0.054	0.084	4.755	0.000	Supported

### 5.4 Test for mediation

We used a bootstrapping approach to examine the mediating effects of different motivation types between perceived advertising value and green purchase intention. As shown in Table 7, at the 1% confidence level, the mediating effect of external regulation, identified regulation, integrated regulation and intrinsic motivation were significant, while the mediating effect of introjected regulation between perceived green advertising value and green purchase intention is insignificant (p-value < 0.01). The results of the mediation analysis indicate that perceived green advertising had significant indirect effects on green purchase intention via different motivation types (external regulation, identified regulation, integrated regulation and intrinsic motivation). Integrated regulation and intrinsic motivation have stronger mediating effects compared to others.

**Table 7:** Mediation Analysis.

Path	Original sample	Standard deviation	*t*-statistics	*p*-values	Mediating effect
PAV–> EXT–> GPI	0.063	0.020	3.206	0.001	Significant
PAV–> INTRO–> GPI	−0.021	0.017	1.208	0.227	Insignificant
PAV–> IDEN–> GPI	0.114	0.037	3.048	0.002	Significant
PAV–> INTG–> GPI	0.285	0.036	7.853	0.000	Significant
PAV–> IN–> GPI	0.171	0.038	4.490	0.000	Significant

## 6 Discussion

Our results indicate that the perceived value of green advertising enhances both extrinsic and intrinsic motivations, and those motivations increase consumers' intention to purchase green products, except for introjected regulation. From the significance of mediators, the findings highlight the importance of various motivational factors in mediating the relationships between perceived green advertising value and green purchase intention. Specifically, intrinsic motivation and integrated regulation emerged as particularly strong mediators, suggesting that effective green advertising should focus on fostering personal connections to environmental issues.

First, our findings show that perceived green advertising value influences various consumer motivation types toward green purchasing intention, supporting H1 to H5. Effective green advertising strongly enhances both intrinsic and extrinsic motivations. Also, our results strengthen green advertising value's role in linking perceived values to different forms of motivation. Specifically, when consumers perceive green advertisements as valuable, this fosters various motivational types, including external regulation (social approval), introjected regulation (guilt or obligation), identified regulation (personal values), integrated regulation (identity alignment), and intrinsic motivation (personal satisfaction). The results underscore the importance of perceived green advertising value in shaping consumer motivations, highlighting that advertisements that resonate with consumers' values can significantly enhance their engagement with sustainable products.

Second, regarding motivation's impact on green purchase intention, the results indicate the lack of impacts from introjected regulation (H7 rejected). One possible explanation for this finding is that introjected regulation, which is characterized by feelings of guilt or shame relating to not engaging in pro-environmental behaviors (Pelletier et al., 1998), may not offer a strong enough motivational force to influence purchasing decisions. While introjected regulation can evoke feelings that raise environmental concern, it often fails to translate these feelings into actual purchasing behaviors (Pelletier and Sharp, 2008), particularly when consumers question whether a green product or service is genuinely beneficial or valuable. Further, growing exposure to greenwashing has fostered widespread skepticism and eco-fatigue. Our findings are consistent with evidence that more experienced green consumers tend to react to green advertising with heightened skepticism. Carrete et al. (2023) show that higher levels of green purchasing are associated with lower credibility of green advertising, especially for low-cost products where environmental benefits are easy to verify. This pattern matches with Japanese cultural norms, which emphasize interpersonal harmony, norm conformity, and long-term societal well-being over explicit, individualized guilt as a primary driver of action. In a high-context, collectivistic society like Japan, pro-environmental behaviors are often embedded in shared social expectations, making norm-aligned and personally endorsed motives (identified and intrinsic regulation) more culturally congruent than individualized guilt. Consequently, introjected regulation exerts limited influence on green purchase intention, whereas more autonomous forms of motivation—with stronger cultural and cognitive support—are more likely to translate perceived green advertising value into purchase intention. This finding also aligns with Ng et al. (2024) that, even if making a purchase can avoid feelings of guilt (introjected regulation), consumers still lack motivation to act.

On the other hand, the results show that identified and integrated regulation positively influences green purchase intention, supporting H8 and H9 and aligning with previous findings (Chen et al., 2016; Ng et al., 2024). Integrated regulation, the strongest predictor (H9), suggests that when consumers view green purchasing as part of their identity or lifestyle, they are more likely to act. Intrinsic motivation also increases purchase intention, supporting H10 and aligning with studies showing that personal satisfaction drives pro-environmental behaviors (Ejelöv et al., 2022; Duong et al., 2023). These patterns are compatible with Japanese cultural characteristics. Japan's high long-term orientation supports future-oriented investments in environmental protection, making it culturally coherent for consumers to internalize green purchasing as a personally meaningful, long-range commitment rather than a short-term, guilt-driven response.

Further, external regulation (H6) is positively associated with green purchase intention, though less strongly than intrinsic and integrated motivations; this is inconsistent with Ng et al. (2024). Possible reasons for this result may come from the research sample and the cultural context. Ng et al. (2024) examined motivation types' impacts on Chinese consumers' green behaviors, with more than 70% respondents under the age of 35; whereas we collected data from Japanese consumers, with more than 70% respondents being over the age of 35. In Japanese context, high uncertainty avoidance leads to conformity with norms as low-risk behavior. Green consumption becomes habitual group compliance (everyone recycles, so I do), aligning with external regulation. While external regulation can encourage green purchases through rewards or recognition, its weaker influence suggests that marketers should combine social norms and peer influences with appeals to personal values and benefits.

## 7 Conclusions

### 7.1 Theoretical implications

We applied the S-O-R model as a theoretical mechanism to deepen the understanding of green advertising, contributing to the advertising literature by positing that perceived green advertising value (*S*) acts as a key factor enhancing consumers' motivation (*O*), which in turn drives their purchase intention (*R*). By investigating how different motivation types mediate the relationship between perceived green advertising value and green purchase intention, we have revealed the motivational pathways that link perceived green advertising value to purchase intention, showing that higher perceived value strengthens intrinsic motivation and integrated regulation, thereby increasing sustainable purchase intention.

We have also extended SDT to the green consumption context by examining how perceived green advertising value shapes different motivation types and green purchase intention. We have highlighted the key roles of integrated regulation and intrinsic motivation, such as personal satisfaction and enjoyment, in promoting pro-environmental behaviors, consistent with previous studies (Wang et al., 2021; Lima et al., 2024; Ng et al., 2024). Unlike Ng et al. (2024), however, introjected regulation showed no significant effect, suggesting that the differences in demographics and culture may influence how consumers respond to social approval in sustainable contexts. This application of SDT enriches the theoretical framework by demonstrating that both intrinsic and extrinsic motivations can coexist and can interact to influence consumer decisions in the green market. Moreover, our study moves the value–motivation link upstream: rather than assuming value is formed mainly at the product experience level (Ng et al., 2024), value can already be generated at the advertising stage and then internalized via different regulation types, suggesting that SDT mechanisms can begin to operate earlier in the consumer journey.

Our study aligns with and expands on the literature that explores the interplays between intrinsic and extrinsic motivations in driving pro-environmental behaviors. In addition, having been conducted in Japan, this study brings valuable cross-cultural insights to the green consumption research. While most of the SDT-based green consumption studies focus on product or general green behavior, often in emerging markets, this study extends the boundary conditions of SDT-based green consumption theory by showing that the mechanism of value-motivation-intention applies to a mature consumption context and to advertising-based stimuli. The difference in our finding on external regulation's role and the finding of Ng et al. (2024), a study done in China, suggests that different cultures may lead to variations in the motivations for green behaviors, calling for further investigation.

In summary, this study reveals three novel mechanisms: perceived green advertising value functions as a motivational trigger within S-O-R rather than a direct driver of intention, demonstrating that the value–motivation–intention mechanism already operates at the advertising stage rather than only at the product stage; a self-determination level-based motivation filter ensures that autonomous motivations (identified, integrated, intrinsic) effectively transmit this value to purchase intention, whereas guilt-based introjected regulation does not; and a dual pathway in controlled motivations emerges in Japanese context, where external regulation plays a weaker but positive role and introjected regulation is non-significant, indicating differentiated contributions of norm-based and value-based motives to green purchasing.

### 7.2 Practical implications

This research has practical implications for green marketers. Regarding strategic marketing approaches, effective strategies should emphasize emotional connections and personal benefits rather than guilt or external pressure. By emphasizing the intrinsic rewards of eco-friendly purchases—such as personal satisfaction, contributions to environmental sustainability, and alignment with one's identity—firms should foster stronger consumer engagement and loyalty. Marketers should also enhance consumers' perceived green advertising value, because it strongly influences the motivation for green consumption. This can be achieved by making advertisements informative, engaging and credible while minimizing irritation; for instance, by providing clear environmental benefits, using appealing visuals or narratives, levering trusted endorsements, and avoiding overly aggressive tactics.

### 7.3 Limitations and future research

Despite the comprehensive findings and implications, this study has limitations. First, as the sample consisted solely of participants from Japan, the findings may be limited to Japanese society. We found differences between different motivation types' influences on green purchase intention, unlike the study done in China by Ng et al. (2024). Further research may recruit participants from various countries and different cultural backgrounds to explore how motivations for green consumption behavior differ across cultures. Second, more than 70% of our respondents were over the age of 35. Some studies have investigated Generation Z, with their more pragmatic spending habits and strong inclination toward personalized and sustainable products (Dragolea et al., 2023; Elgammal et al., 2024). Researchers are encouraged to focus on younger demographics to explore their perspectives and behaviors regarding green advertising and sustainable consumption. Understanding younger consumers' attitudes may provide valuable insights into how marketing strategies can be tailored to effectively engage this group. Third, this study is cross-sectional, using convenience sampling and data collection at a single time point. Future studies could consider longitudinal designs to capture changes in consumers' green consumption behavior and their perceptions of green advertising over time.

## Data Availability

The original contributions presented in the study are included in the article/supplementary material, further inquiries can be directed to the corresponding author.
